# A power-sharing perspective on employees’ participatory influence over organizational interventions: conceptual explorations

**DOI:** 10.3389/fpsyg.2023.1185735

**Published:** 2023-07-13

**Authors:** Robert Lundmark

**Affiliations:** Department of Psychology, Umeå University, Umeå, Sweden

**Keywords:** autocratic, consultation, delegation, participation, empowerment, context

## Abstract

A participatory approach is widely recommended for organizational interventions aiming to improve employee well-being. Employees’ participatory influence over organizational interventions implies that managers share power over decisions concerning the design and/or implementation of those interventions. However, a power-sharing perspective is generally missing in organizational intervention literature. The aim of this paper is therefore broaden the picture of the mechanisms that influence, more or less, participatory processes by conceptually exploring this missing part to the puzzle. These conceptual explorations departs from both an empowerment and a contingency perspective and results in six propositions on what to consider in terms of power-sharing strategies, reach, amount, scope, culture and capacity. Implications for research, as well as for organizations and practitioners interested in occupational health improvements, are then discussed. Especially, the importance of aligning power-sharing forms with the needs of the participating employees, and taking factors that can facilitate or hinder the power-sharing process into consideration, are stressed. The importance of training managers in power-sharing practices and supporting a participatory process is also highlighted.

## Introduction

Organizational interventions focus on change in how work is organized, designed, and managed to improve the well-being of employees (Nielsen et al., 2010). By targeting improvements in factors that contribute to the work environment, organizational interventions have the potential to benefit many at the same time, over time. Therefore, they are generally recommended for improving employee well-being (e.g., Kelloway and Day, 2005; Nielsen and Noblet, 2018). Organizational interventions often address problems emerging from employees’ concerns about their work environments (Tvedt and Saksvik, 2012). Consequently, employee participation in the development of efficient and effective solutions to identified problems is a natural next step. For example, in a recent conceptual paper aiming to identify important principles on what to consider when designing, implementing, and evaluating organizational interventions, employee participation was the first principle (von Thiele Schwarz et al., 2021). Thus, systematic approaches to solving identified work-environment problems unanimously highlight employee participation (Nielsen and Abildgaard, 2013; Fridrich et al., 2015; von Thiele Schwarz et al., 2016).

Beyond improving the identification of problems and solutions, employee participation can also help contextualize intervention activities to improve their fit into ongoing organizational operations. It can also help align these activities with the needs of those involved (von Thiele Schwarz et al., 2016; Lundmark et al., 2021). Employee participation has even been suggested as a possible intervention in itself because the empowerment experience that comes with active participation contributes to improved employee well-being (Theorell, 2003). However, although participation is widely recommended, there is little guidance on what it really means in terms of whom should participate, to what extent, and how it can be achieved.

The aim of this paper is to start filling this gap by focusing on how different levels of employee participation can emerge from different forms of power sharing. The aim is also to elaborate on five factors known to influence the power sharing-participation process: Reach (i.e., who are participating?; Lehmann et al., 2022), Amount (i.e., how much power is shared?; Lee et al., 2017), Scope (i.e., what kind of decisions are shared?; Richardson et al., 2021), Culture (i.e., where is the intervention taking place?; Tvedt and Saksvik, 2012), and Capacity (i.e., what are the prerequisites?; Coffeng et al., 2021). Thereby, starting to fill the gap on what organizations should consider when involving employees in the design and implementation of organizational interventions. Hence, it concentrates on the control over decisions part of the participation process, explicating the ways organizations may foster participation during interventions, and what they can expect from doing so. Thereby, it adds a missing part to the puzzle to the understanding of the power sharing—participation process. As of now, only advocating participation without proposing how this can be achieved (i.e., in terms of power sharing) increases the risk of making participation in organizational interventions nothing but a fancy phrase. In addition, participation without the appropriate mandate to influence decisions may lead to outcomes adverse to what is desired, such as resistance to change instead of empowerment. By highlighting the roles of potential approaches and boundary conditions, guidance for organizations on what to consider is provided.

The paper is structured so that it first describes two commonly used points of departure for examining power sharing: empowerment theory and contingency theory. Thereafter, different forms of power sharing are clarified, as is what can be expected from them in terms of employee participation during organizational interventions. Current knowledge on the influence of different conditions is depicted, and from that, propositions are made to introduce and guide the understanding of employees’ participatory influence over organizational interventions from a power-sharing perspective. Finally, implications for research and practice are discussed.

## Two points of departure: empowerment and contingency theories

Empowerment and contingency theories are widely applied in literature to explicate the power-sharing–participation relationship and its outcomes (Cheong et al., 2019). Although representing different perspectives, they can both contribute to the understanding of power sharing as a way to enable employee participation during organizational interventions.

Psychological empowerment (Spreitzer, 1995) is a motivational state comprised of meaning (i.e., alignment between one’s own ideals and the requirements of one’s work role), competence (i.e., belief in one’s capability to perform well within a work role), self-determination (i.e., autonomy in the performance of one’s work role), and impact (i.e., possibilities to influence outcomes at work). Psychological empowerment has been positively associated with an extensive number of desirable employee and team well-being and performance outcomes and found to be a way of promoting democracy at work (Seibert et al., 2011). These cognitions echo an active participatory orientation to decision-making in which employees are interested in and able to form their work roles and influence the context of those roles. Hence, a participatory approach to decision-making is an important antecedent to employee empowerment (on both the individual and group levels) that, in turn, can be seen as a mechanism for producing beneficial employee and team outcomes (Seibert et al., 2011).

Psychological empowerment has also been found to mediate the relationship between job crafting (i.e., alterations to the job made through employee initiative; Tims et al., 2016) and employee outcomes such as job performance (Maden-Eyiusta and Alten, 2021). Thus, from an empowerment perspective, employees’ active participation in crafting organizational interventions can be seen as beneficial in itself. That is, rather than a means to an end, high levels of employee participation in the design and implementation of organizational interventions are part of the end. However, to achieve a sufficient level of such participation practices, a certain degree of power sharing is necessary (Abildgaard et al., 2020).

Adding to this perspective is that with more power over decisions, employees have greater chances of controlling which activities to implement, what tasks to perform, and how to perform these tasks based on their competences and needs (Biron and Bamberger, 2011). It has also been suggested that allowing employees to act within their competencies enhances their sense of control. In turn, this can buffer the detrimental effects of increased demands on well-being and performance (Van Yperen and Hagedoorn, 2003). Based on these arguments, creating a fit between employees’ competences and needs and an organizational intervention is often highlighted (Lundmark et al., 2021). To create such an intervention fit, employees need to be active participants rather than passive recipients in the process of creating and implementing organizational interventions (von Thiele Schwarz et al., 2016).

A contingent perspective instead emphasizes that the effectiveness of different forms of power sharing is dependent on specific situational factors (Vroom and Jago, 2007; Oc, 2018). In line with this contextual focus, the conditions under which managers and employees interact and under which organizational interventions take place are often stressed (e.g., Lundmark et al., 2020). Taking a contingent perspective on organizational interventions involves asking questions such as

1. Where is this taking place (e.g., in terms of country and culture)?
2. Who is involved (e.g., composition of those involved)?
3. When (e.g., during turbulent times)?

It also involves appraising aspects of the work at hand in terms of job characteristics related to the task and any social, physical, or temporal issues (Oc, 2018). Specific aspects can also combine to produce outcomes; for example, time spent on discussions in teams may be related to team climate, and team climate may, in turn, influence time spent on discussions. Furthermore, these aspects are seen both as a potential antecedent to the power-sharing process (e.g., determining what form of power sharing is possible) and as a moderator in the process (e.g., determining the effect of different power-sharing forms; Richardson et al., 2021).

From this viewpoint, a form of power sharing (e.g., shared decision-making) that is effective in one situation may prove totally ineffective in a different situation (Schweiger and Leana, 1986; Vroom and Jago, 2007). Hence, managers’ power sharing with employees should be adapted to fit the circumstances of each specific situation. Although participation here can be understood as a means rather than an end, Vroom (2003) clearly stated that apart from decision effectiveness, employee development should be considered when choosing the power-sharing–participation strategy. Thus, if employee development is of the essence (e.g., viewed as a goal), then this can help determine employees’ levels of participation in decision-making processes.

**Figure 1:**
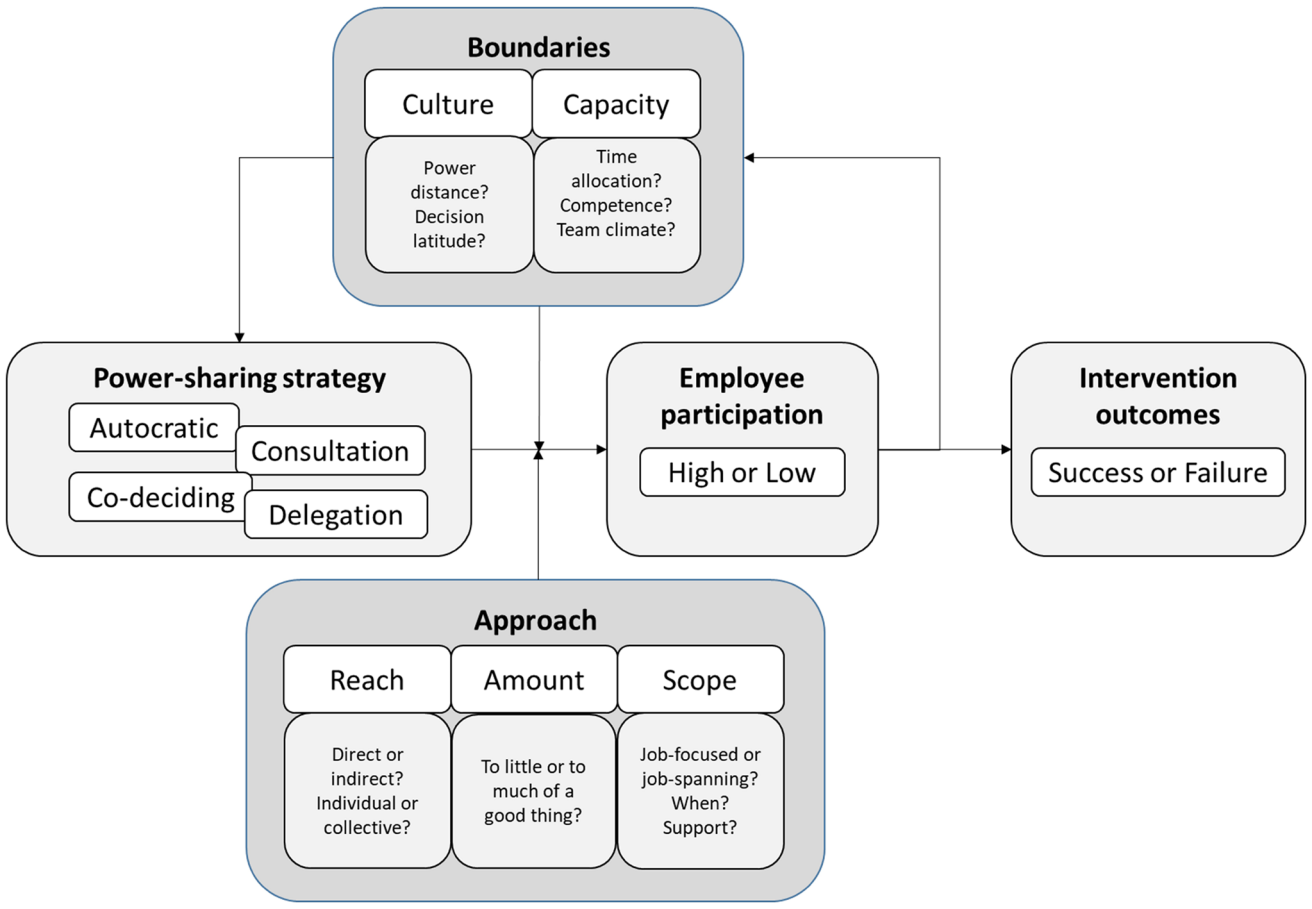
The power-sharing–participation–outcome process.

In the following sections, power sharing during organizational interventions, its outcomes, and five potentially influential aspects that influence the power-sharing–participation–outcome process (see Figure 1) are deliberated. The aspects considered in this paper are not meant to be exclusive or exhaustive but rather a starting point for further explorations.

## Power sharing strategies

As Abildgaard et al. (2020) pointed out, there is a difference between *participating in* intervention activities and having *participatory influence over* decisions on what kind of intervention activities are suitable. However, whether employees participate only marginally by taking part in intervention activities or exert a participatory influence over interventions is, in turn, ultimately a question of power sharing in various degrees (Hollander and Offermann, 1990). In other words, employee participation is directly dependent upon managers’ sharing of power in some form, and the degree of power sharing will accordingly affect the level of employee participation.

Power sharing as a way to enable participation can be seen as a continuum reflecting the amount of power being shared (Vroom, 2003; Biron and Bamberger, 2011). At one end is *autocratic decision-making*, where employees have no influence over decisions. At the other end is *delegation*, where employees are allowed to make decisions on their own (i.e., power is distributed rather than shared; Hollander and Offermann, 1990). In between autocratic decision-making and delegation are power sharing through *consultation* and *shared decision-making*, in which employees are asked for opinions before decisions are made or are invited to codecide (Vroom, 2003).

*Autocratic* decision-making involves no involvement of employees in decisions and can hence be described only in terms of obligation to partake in activities according to premade decisions (Hollander and Offermann, 1990). Managers thus announce decisions for employees to heed (Hollander and Offermann, 1990; Vroom, 2003). This form of decision-making allows no employee participation in determining the goals, content, and processes of an organizational intervention. Such minimal employee influence is associated mainly with interventions where these components are preset (Abildgaard et al., 2020). In other words, employee participation is understood primarily in terms of fidelity to and/or compliance with an intervention protocol.

*Consultation* involves managers asking for employees’ ideas or suggestions (Tangirala and Ramanujam, 2012). Here, employees are given the possibility to influence decisions prospectively and indirectly by giving their views on matters. If managers listen to employee concerns, consultation can enhance the employees’ sense of control and confidence in their abilities to influence decisions (Tangirala and Ramanujam, 2012). From an organizational intervention perspective, employee involvement in decisions on a consultation level has been highlighted as a minimal form of participation (Abildgaard et al., 2020). Even if intervention goals are preset, consultation allows for the creation of a better fit between the intervention and the employees’ concrete needs and competences and between the intervention and the context in which it takes place (Randall and Nielsen, 2012). For example, employees can contribute with suggestions for how activities can be adapted or adjusted and can provide input on the timing of different activities (von Thiele Schwarz et al., 2016).

*Shared* decision-making occurs when employees are engaged in specific decisions on terms equal to those of their managers (and, in some cases, other stakeholders; Hollander and Offermann, 1990). Here, rather than having decisions delivered to them, the process is viewed as a joint venture for creating value for employees and their organizations (Payne et al., 2008). In this form of decision-making, managers strive for concurrence on decisions, and their role is to act as facilitators who define problems and boundaries (Vroom, 2003). Employee participation in the co-creation of an organizational intervention is often recommended (von Thiele Schwarz et al., 2016). Beyond the benefits in terms of intervention fit, increased employee empowerment is often underscored as an instrument for implementation success (e.g., by contributing to higher levels of engagement and attendance in activities; Nielsen, 2013). Because an increase in employees’ control over decisions may contribute to their development, well-being, and productivity, shared decision-making has been suggested as an intervention, or intervention goal, in itself (Theorell, 2003).

*Delegation* is suggested as the power-sharing practice that can enable employee empowerment to the highest degree (Richardson et al., 2021). In delegation, managers allocate decision-making authority to employees as opposed to situations where leaders make decisions either alone or jointly (Cheong et al., 2019). Because delegation emphasizes employees’ autonomy and enhanced responsibilities, it is also seen as the direct opposite of autocratic decision-making. Thus, delegation implies moving the authority from one level to another—distributing rather than sharing the power (Leana, 1986). Delegation is highly associated with empowerment and employee development and clearly focuses on participation as a goal in itself (Vroom, 2003; Biron and Bamberger, 2011). Despite this, intervention evaluation studies have seldom suggested delegation as a strategy for enhancing employee participation (Abildgaard et al., 2020). This may be due to the work environment statutes bestowed upon managers, making it necessary for them to retain some level of control over decisions concerning intervention designs. At the same time, a bottom-up approach, where employees initiate and suggest interventions, has been highlighted as a token of true organizational interventions (Tvedt and Saksvik, 2012).

In practice, a combination of power-sharing approaches may be, and often are, used. For example, senior management may decide autocratically upon the focus of the intervention (e.g., redistributing workload), but consult and share decisions with employees on how and when the change should be implemented, and delegate responsibility for its implementation. Such combined power-sharing approaches can be time saving initially, but also risk missing the target, as the content may not match what employees’ perceive as their primary needs (e.g., Biron et al., 2010). Shifting power-sharing strategies can also contribute to employees’ perceiving their managers intentions as confusing, and thus lead to a time-consuming sense-making process of what mandates exists and what is expected from employees (Schilling et al., 2022).

Proposition 1: Employee participation during organizational interventions is dependent upon managers’ power-sharing strategy. Organizations should explicitly consider what strategy is most appropriate to use given what they wish to achieve in terms of participation, and ultimately intervention outcomes.

## Approaches to power sharing

### Reach of power sharing

With the exception of autocratic decision-making, all other power-sharing forms can be performed in either a dyadic (manger and employee) or collective (manager and group) manner (Vroom, 2000). In practice, power-sharing practices, especially delegation, generally seem to be more commonly performed on an individual level (Richardson et al., 2021). A reason for this may be that sharing power on a group/collective level demands sufficient time and involves a greater likelihood of disagreements (Vroom, 2003). Additionally, shared decision-making and delegation can be viewed as more delicate and risky than other forms of power sharing because they involve the consideration of more factors (e.g., employee competence and possibilities for job expansion; Richardson et al., 2021). Therefore, managers are more likely to choose employees that they perceive as approachable when distributing power (Leana, 1986). In contrast, power sharing on an individual level during organizational interventions is seldom recommended. Instead, involving all targeted employees is often emphasized (Lehmann et al., 2022). Evidence also suggests that a collective participation process is more effective since it contributes to increased engagement and better team functioning, and thereby influences outcomes to a higher extent (Nielsen et al., 2021).

Representative participation in decision-making is also a common phenomenon (Helland et al., 2021). Representative participation in decision-making (i.e., indirect) can be seen as power sharing on an individual level, even though the representative may have involved others before engaging in the decision-making process. For example, a health and safety officer can act as a representative for the employee collective. A representative can participate in the decision-making process to a greater or lesser extent. In turn, they can also involve the employee collective in the process to a greater or lesser extent (Helland et al., 2021). From a managerial perspective, this may be considered a preferable option, especially in large-scale interventions conducted in large organizations, because it helps reduce time, costs, and logistical problems.

Still, from the perspective of empowerment and democracy at work, indirect involvement may reduce the chances of achieving the beneficial employee outcomes that could be expected from being a direct part of a shared decision-making process. In addition, the success of power sharing through representatives likely depends upon whether the representative involves other employees and whether the representative is viewed appropriately as such (Abildgaard et al., 2020). As Lehmann et al. (2022) has shown, direct participating employees are more likely to experience improvement in intervention outcomes. Contrary, employees that participate indirectly (i.e., through a representative) not only benefit less but can also experience deterioration in intervention outcomes. Implying that not being able to participate directly could have a worsening effect. As the fit of an intervention with employee needs also influences intervention outcomes (Lundmark et al., 2018), indirect or a low degree of direct participation in decisions likely reduces possibilities for such alignment, and thus risk missing targeted objectives.

Power sharing is mainly described in literature as phenomenon between first line managers and employees (Vroom, 2003). Line managers are also focused upon in organizational intervention studies, as they often are the ones with the responsibility to transform plans into actions, communicate and follow-up change (Lundmark et al., 2020). However, this suggests that line mangers at some level have a mandate from senior management to craft changes together with employees. Line mangers’ prerequisites in terms of such mandate when it comes to organizational intervention initiatives has seldom been discussed. Such trickle-down effects (i.e., from senior management to line managers to employees), have however been concluded important for understanding empowering processes in leadership studies (Byun et al., 2020). Thus, aligning power-sharing processes across organizational levels may also be an important aspect to consider.

Proposition 2: Power-sharing that involves all participating employees, and that is aligned across organizational levels, stand a greater chance of reaching high positive impact on employee participation and consequently also on intervention outcomes.

### Amount of power sharing

Too little or too much of a good thing refers to the mechanism that ordinarily produces beneficial outcomes (i.e., in this case, power sharing; Pierce and Aguinis, 2013). That too little power sharing can be detrimental is perhaps no surprise; however, research also has suggested that too much, in the long run, can produce undesirable employee outcomes (Pierce and Aguinis, 2013). Too little power sharing in terms of autocratic decision-making has been concluded to be detrimental to employees because it goes against their basic needs for autonomy and affects their possibilities for growth and development (Theorell, 2003). Employees who lack influence over decision-making can thus feel alienated and withdraw from participating in activities (Tangirala and Ramanujam, 2012).

Similarly, it has been argued that if a manager possesses all the power, they risk being overwhelmed by the decisions they need to make, and employees are frustrated by being hindered or slowed down when managers cannot make timely decisions (Theorell, 2003). Others have suggested that under certain conditions, participation may pose greater risks than gains for decision quality. For example, when there is time pressure and/or a high risk of destructive conflicts, managers may have no alternative but to make decisions on their own. Granted that they have sufficient competence to make decisions, it may therefore be a viable option (Vroom, 2003).

On the contrary, consultation is often viewed as beneficial for employee empowerment (Tangirala and Ramanujam, 2012). However, employees’ contributions are only put into practice if the manager finds them appropriate. Therefore, consultation risks being viewed as power sharing on a pseudo-level, with managers seeking acceptance and justification for their own decisions rather than employee participation in decisions (Biron and Bamberger, 2011). In the long run, employees’ unfulfilled expectations of having their suggestions and ideas accepted may instead discourage them from such participatory practices (Hollander and Offermann, 1990). Shared and delegated powers over decisions are clearly participatory approaches to decision-making that have possibilities to contribute to effective and efficient decisions and are associated with employee empowerment (Cheong et al., 2019).

However, these approaches are also associated with risks (Norris et al., 2021). It appears that with prolonged exposure, the positive effects of such empowerment disappears over time. This inverted U-shaped relationship between power-sharing actions and employee empowerment has been observed in several studies (e.g., Lee et al., 2017; Richardson et al., 2021). There are also studies showing a direct relation between high degrees of power sharing and unfavorable employee outcomes. For example, Norris et al. (2021) showed that ambitions to empower employees through delegation could instead be perceived as a manager’s way of withdrawing from their managerial responsibilities by passing over unwanted tasks, increasing employee resistance.

Additionally, having more power also means having more responsibilities and obligations to participate in extra activities outside ordinary tasks. Employees’ performances in these activities may also to a greater extent become exposed and targeted for critique (Vroom, 2003). As managerial and employees roles are being blurred, chances of role ambiguity increases (Richardson et al., 2021). Thus, employees can become accountable over areas for which they have less expertise and experience, and thus exceed their capabilities. Such extra tasks can also be perceived by employees as illegitimate (i.e., unnecessary or unreasonable) given their designated roles (Björk et al., 2013).

Besides positive outcomes, these participatory approaches to power sharing can therefore have undesirable effects. Rather than empowering, gained power may develop into a burden when employees feel that they cannot meet the challenge or when they see it as unreasonable for them to manage (Cheong et al., 2019; Rosen et al., 2020). That is, having too much to say over matters in which one does not have sufficient competence or support may not always be empowering or developing; it can instead result in stress and depletion (Björk et al., 2013; Rosen et al., 2020). Although this phenomenon is less well documented in organizational intervention literature, there are examples of how employee readiness for participation in interventions influences their perception of managers’ behavioral strategy (e.g., Lundmark et al., 2020).

Proposition 3: The level and duration of power sharing practices should be balanced against employee prerequisites so that participation becomes empowering rather than a burden.

### Scope of power sharing

In addition to considering the form and balance of participation, it is also important to consider the actual scope of the decisions employees are participating in Biron and Bamberger (2011). Richardson et al. (2021) distinguished between two forms of decisions that employees are involved in: job-focused (i.e., core tasks and how and when they are performed) and job-spanning (i.e., strategic, administrative, or operational challenges that require taking over managerial tasks). When the latter occurs, employees’ roles are not just enlarged but enriched, and a ground for growth and deep empowerment (as opposed to surface empowerment) is more likely to be established (Biron and Bamberger, 2011; Richardson et al., 2021).

Findings have indicated that job-spanning consultation is more empowering than job-focused delegation, and that job-focused consultation is negatively related to employee perceptions of empowerment (Richardson et al., 2021). In organizational intervention literature, employee participation has been discussed in terms of a decision on the content and/or process of an intervention (Abildgaard et al., 2020). When employees are given influence over the content and process of an intervention, they, by definition, have a say in what areas of work should be targeted, what the goal of the intervention should be, and what activities should be included. Such decisions could be considered job-spanning. In contrast, interventions with predefined goals, content, and activities have fewer job-spanning decisions that need to be made and therefore leave room mainly for engaging in job-focused decisions.

Participating on decisions concerning the content also means that when power sharing takes place seems to matters. Power sharing at the planning stage has been shown to promote participation throughout the implementation and sustainment of the intervention. It also positively influences intervention outcomes, especially when combined with supportive actions from managers (e.g., following up on delegated tasks; Tafvelin et al., 2019). Thus, from an empowering perspective (Cheong et al., 2019), the cost in time to involve employees at an early stage in job-spanning decision-making may be returned later on in the process as it may enhance motivation and satisfaction, and reduce time spent later on in the dissemination of the intervention. These results are also consistent with suggestions on the importance of involving employees at an early stage to create a fit between intervention content, the context where the intervention takes place, and the people involved (Lundmark et al., 2018).

Proposition 4: Power sharing to promote participation during organizational interventions is important from the start (i.e., planning of the intervention) and should preferably be combined with managers’ support throughout the process. Power sharing should preferably involve job-spanning, rather than job-focused, decisions to create a better intervention fit.

## Boundary conditions

### Power sharing culture

Organizational intervention frameworks stress that it is vital to consider where an intervention takes place (e.g., in terms of its cultural context); at the same time, they consistently advocate highly participatory approaches (e.g., Nielsen and Abildgaard, 2013). However, the Western cultural context where these frameworks were developed and evidence was gathered for their support is rather heterogenic (Tvedt and Saksvik, 2012). For example, Irastorza et al. (2016) found that whether employees in Europe had a say in the designs of interventions focusing on the psychosocial work environment differed depending by country, with Nordic country employees having the most influence on such matters. In Nordic countries, self-governed work groups with egalitarian cultures are common and may thus provide favorable platforms for sharing power and for conducting organizational interventions using a participatory approach (Theorell, 2003). Conversely, in previous attempts, applying frameworks developed in cultures other than the ones addressed has posed problems due to the poorer fit with views of power sharing (Tvedt and Saksvik, 2012).

Hofstede (1980) introduced the concept of power distance as an important distinguishing determinant of management in different countries. Hofstede (1980) argued that distance in power, “the extent to which a society accepts the fact that power in institutions are distributed unequally” (p. 6), could explain whether an autocratic decision-making or a participatory approach is present. Thus, the concept postulates that organizations in countries or cultures with high power distances, more autocratic management styles are generally preferred. In contrast, in countries and cultures with low power distances, more participatory management styles are generally favored (Javidan et al., 2016). Similarly, Triandis (1994) concluded that the orientation of cultural values in a country would determine its power-sharing profile. Individualist countries tend to put more emphasis on freedom and challenges, whereas collective cultures favor security, obedience, group harmony, and duty (Triandis, 1994). For example, Newman and Nollen (1996) studied how power-sharing practices improved the profitability of work units in different countries. They found high degrees of power sharing were effective in countries with relatively low power distances but did not affect profitability in cultures with high power distances. In other words, different power-sharing practices are contingent upon what is culturally acceptable, which also suggests that they are more or less effective depending on where an intervention takes place.

However, from the perspective of empowerment and democracy at work, national culture is argued to be of less importance, and a non-participative approach is viewed as problematic regardless of where it appears. Autocracy simply stands in the way of developing employee autonomy and control (Rothschild, 2000). In turn, evidence has suggested that a lack of autonomy and control constitute a link to poor employee well-being and performance (Theorell, 2003). Thus, given sufficient time to introduce a participatory approach, advocating heightened employee latitude in decision-making can be an intervention in itself, even in cultures with high power distances (Budd et al., 2018). Democratization of the workplace, through genuine employee participation in decisions, can thus be seen as a profound goal to strive for under any circumstances as an ethical imperative that is (at least long-term) also sound for employee well-being and performance (Sashkin, 1984; Foley and Polanyi, 2006). Additionally, instead of viewing power sharing during organizational interventions as an effect of democratic culture, promoting democracy through participation can have a cascading effect that inspires change in a wider organizational and societal democratic process (Budd et al., 2018).

Proposition 5: When considering power-sharing strategies for designing and implementing organizational interventions, power distance culture should be taken into account.

### Capacity for power sharing

In decision-making literature, time is often considered a vital factor in determining the form of power-sharing practice (e.g., Richardson et al., 2021). Engaging employees to participate in the decision-making process naturally takes time, increasingly so based on the amount of power being shared. For example, a shared decision process with ambitions to achieve consensus is likely time consuming, especially if there are conflicting opinions. Similarly, some decisions may have short deadlines or may be connected to a crisis, leaving little room for employee participation in decision-making (Vroom, 2003). Highly participatory forms of power sharing that are not accompanied by sufficient participative time may thus be counterproductive. Managers under time pressures may have to switch to less participatory forms or end up with low-quality decisions because the process is rushed. Rather than contributing to the empowerment and development of employees, the lack of fit of time with the process may instead be experienced as a stressor and may contribute to adverse outcomes (Björk et al., 2013). The success of organizational interventions in which participation is deemed a goal in itself, and thus highly participatory forms of power sharing are necessities, are therefore likely dependent upon having ample time to process decisions.

The amount of time needed for different forms of power sharing may, in turn, be contingent upon other factors, for example, managers and employees’ readiness for participating in shared and distributed decision processes (Yang, 2015). At lower stages of readiness, autocratic decision-making can initially outperform more participatory forms in terms of time and quality, because the individual employees and teams are uncertain about what is expected of them (Lorinkova et al., 2013). Competence (i.e., in terms of knowledge and experience) among both managers and employees is a vital ingredient for readiness, and has been concluded to be significant for high-quality decision outcomes in power-sharing processes (Vroom, 2003). Competence here refers to both procedural and content competence and can be seen as a central component for both managers and employees’ readiness for intervention participation. That is, competence in exercising power to different degrees (e.g., knowledge of the delegation process) and competence concerning the content of decisions (e.g., organizational intervention designs and activities). A high level of trust in a manager’s competence contributes to power sharing being perceived as propitious by employees (Norris et al., 2021).

Conversely, employee perceptions of low competence in their managers tend to result in adverse evaluations of their power-sharing practices (Norris et al., 2021). Similarly, managers who perceive that employees lack competence and trustworthiness will be reluctant to share power with them because the quality of decisions may be reduced. In such cases, autocratic decision-making or consultation may be more tempting alternatives, especially if time is of the essence (Vroom, 2003). On the other hand, involvement in participatory interventions can be a lesson in itself, and as individuals and teams develop, so does their readiness for effective participation in decision-making. Thus, over time, with increased experience, clarification of roles, and commitment to a shared mission, more time-consuming forms of power sharing can be performed more rapidly and produce higher quality decisions. However, this demands investments in time (Coffeng et al., 2021).

Similarly, team climate (i.e., norms, attitudes, and expectations that are perceived by team members; Schneider, 1990), is often mentioned in conjunction with decision-making processes as a strongly contributing factor for decision effectivity and quality (Coffeng et al., 2021). For example, teams who actively participate in decision-making develop trustful relationships and commitments to the team goals (e.g., Costa et al., 2001). Team climate has been researched both as a mediatory outcome of managers’ power sharing that, in turn, influences the effectiveness and quality of team decisions (Coffeng et al., 2021) and as a moderator that influences the power-sharing–employee behavioral process (Cheong et al., 2019). Conversely, Vroom (2003) suggested that using a participatory approach when competence is low and time is short instead may influence team climate negatively and consequently reduce decision effectivity and quality. From this, a vicious circle can develop, where disagreements and destructive conflicts appear which further reduce a team’s decision-making effectivity and quality, and hinder future ambitions for participatory decision processes (Vroom, 2003).

Proposition 6: For a participatory process to be realized, sufficient capacity for a high degree of power-sharing practices must be in place. For example, in terms of allocated time, managers’ and employees’ competence, and team climate. Over time, participatory power-sharing practices can increase capacities.

## Discussion

The purpose of this paper was to introduce a power-sharing perspective on employees’ participatory influence over organizational interventions. Although preferred ways of power sharing are often implicitly suggested in intervention literature (e.g., by focusing on co-creation), guidance for understanding different forms of power sharing are and what needs to be in place for them to be effective is sparse. In the paper, six propositions are made to sum up the conclusions that can be drawn from the literature. These propositions are intended to help guide researchers and practitioners interested in how power-sharing strategies influence participation, and how different approaches and boundary conditions may influence the power-sharing–participation–intervention outcome process, see Figure 1. Culture and Capacity are here depicted as potential antecedents to the choice of strategy, but also boundary conditions in the relation between power sharing strategy and employee participation. At the same time, as a high degree of employee participation may influence both culture and capacities, they could also be viewed as outcomes of a high degree of employee participation. For example, fostering employee participation may over time improve decision making in teams, and thereby enhance team-climate and decision quality, as well as reduce time for decisions. The three approaches (reach, amount, and scope) function as moderators in the power sharing strategy—employee participation relation, as they can increase or reduce the influence of the different strategies on employee participation.

Two perspectives are clearly present in an examination of power-sharing literature. One suggests that levels of power sharing should depart from analyzing contextual conditions, such as the surrounding culture, what time is given, and the competence of employees (Vroom and Jago, 2007). Here, decision effectivity and quality are often seen as primary outcomes, and employee participation in decisions to various degrees as means for reaching these outcomes (Vroom, 2003). The other perspective suggests that heightened employee latitude in decision-making enhances employee outcomes (i.e., in terms of well-being and performance) and therefore always should be advocated (Theorell, 2003). In recent research, attempts have been made to combine these perspectives (e.g., Biron and Bamberger, 2011; Richardson et al., 2021).

Although the empowerment of employees may have favorable outcomes, it is clear that empowerment processes are also dependent upon conditions, which contribute to sometimes making those processes burdens rather than possibilities for development (Cheong et al., 2019). However, this does not necessarily mean that relying on less empowering forms of power sharing is required. It could instead suggest that clearly stating employee participation in decision-making as part of the goal of an intervention is important, because that will help determine what prerequisites need to be in place before initiating organizational interventions, for example, by allocating a sufficient amount of time given the competence and climate of a team for a participatory process. It could also suggest that organizations to a higher degree should consider what kind decisions are shared (i.e., job-spanning or job-focused) and what support is given to employees exercising allocated power.

As mentioned in the introduction, the aspects considered in this paper are not meant to be exclusive or exhaustive but rather a starting point for further explorations. However, one closely related factor to power sharing that may also be worth considering is leadership. Most leadership theories, implicitly or explicitly, includes features of power sharing strategies (Cheong et al., 2019). For example, specific sets of leadership behaviors focused on the development of employees through challenges (i.e., intellectual stimulation) is a central aspect of Transformational leadership that is closely linked to power sharing (Bass and Riggio, 2006). In Empowering leadership theory (Cheong et al., 2019), a high degree of power sharing (i.e., through delegation) is also considered a central component for achieving high levels of engagement among employees. Contrary, autocratic leadership styles, such as Abusive supervision (Tepper, 2000), are associated with low degrees of power sharing. From this, advocating a constructive leadership style, in general, and specifically in the context of organizational interventions, has been shown beneficial for intervention outcomes (Lundmark et al., 2020).

### Implications and future directions for research

Introducing power sharing as a complementary perspective to employee participation can broaden the understanding of why and when organizational interventions are successful or not. Process evaluations, including assessments of participation, are widely used for answering such questions (Nielsen and Noblet, 2018). They are also used to be able to make adaptions to a process as it evolves (von Thiele Schwarz et al., 2016). By including assessments of how, what, when, and to what degree, managers power share in the intervention process may thus further facilitate the understanding of mechanisms contributing to success or failure. For example, if a participatory approach is used for designing and implementing an organizational intervention, managers’ initiation of co-creation and/or power distribution can be evaluated. This can help explain why participation is present or not and can facilitate problem-solving during implementation if participation is present to a lesser degree than intended. Such assessment tools could likely be adopted from literature on empowering leadership and decision-making (for an overview, see Cheong et al., 2019) and adapted to an intervention context.

Furthermore, researchers could also benefit from considering power sharing in research-driven intervention designs. For example, if the content is more or less predecided, can empowering forms of power sharing in decision-making still be introduced? Can some decisions be performed at a consultation level and others be delegated, and how are such changes in strategies understood? How can the role ambiguity that may come with a role expansion be mitigated? Answering such questions and examining the effects of the power-sharing strategies applied could further help advance the understanding of what is appropriate, for whom, when, and to what extent, in line with calls for a better understanding of the process (Nielsen et al., 2010).

### Practical implications

From a managerial standpoint, knowing that although inviting employees into the participatory decision process may consume time and effort, it can also contribute to improving an intervention’s design and hence its outcomes. It can also have cascading effects, for example, in terms of increasing employee autonomy and control; building positive relationships that improve team climate; aligning individual, team and organizational goals; and enhancing commitment to the organization. Thus, elevating employee latitude in decision-making on designing and implementing organizational interventions contributes both directly and indirectly to achieving the objectives of the intervention. However, managers must balance participatory ambitions with contextual considerations (e.g., the time and competence at hand). If these ambitions do not align with such prerequisites, there is instead a substantial risk of detrimental outcomes (Norris et al., 2021).

In sum, managers should carefully consider the objectives of an intervention and what level of employee participation will contribute to reaching these objectives. They may also want to consider additional gains for the organization by establishing different power-sharing practices (e.g., promoting democracy at work). The objectives must then align with the power-sharing strategies (Vroom and Jago, 1988). If not, employees may experience being misled (e.g., having a say in issues that do not matter or their decisions being neglected), with the potential failure of the intervention and hampered motivation to participate in future initiatives as results. Finally, embarking on a participatory power-sharing process without aligning such a strategy with sufficient contextual prerequisites is a road to failure (Richardson et al., 2021). Hence, assessing the preconditions and influential contingent factors to make sure that they are acknowledged in planning (e.g., in terms of time, activities, and support) is vital.

Considering the complexity of the above, if participatory organizational intervention approaches are to be encouraged, organizations need to train managers in essential power-sharing skills for achieving meaningful participation. Such educational activities must also contribute to managers’ abilities to determine the necessary preconditions to be fulfilled, the conditions and support needed during the intervention’s implementation, and the knowledge needed about the potential pitfalls of the different power-sharing paths. The training of managers should also involve how to shift strategies consciously to avoid too much of a good thing, for example, as Richardson et al. (2021) suggested, to shift between job-focused delegation and job-spanning consultation and delegation to correspond with employees’ needs for both empowerment and control. At the same time, to this in a way that is not perceived as inconsistent may be a challenge, and ways of introducing such strategies could be benefited from simultaneous or joint employee training. For example, training in power-sharing practices could very well be more functional if managers and their teams learn together, which perhaps also can facilitate the transfer of such skills to practice.

## Conclusion

In this study, the concept of power sharing was explored in relation to the designs, implementations, and outcomes of organizational interventions. Although power-sharing practices are determinants of employee participation, an often considered central aspect of organizational interventions, they have seldom been the focus of attention in intervention literature. By departing from a power-sharing perspective, implications that this may have for organizational interventions were conceptually examined. Thereby, this study hopefully contributes to building a platform for future examinations of the power-sharing concept in organizational intervention contexts. From a practitioner viewpoint, understanding the importance of aligning power-sharing forms with participants decision needs is stressed. To achieve such a fit, factors that can facilitate or hinder the power-sharing process must also be considered. Furthermore, managers must be given appropriate training in how to determine and implement different power-sharing strategies, and supplied with adequate support for realizing participatory decision-making practices.

## Author contributions

The author confirms being the sole contributor of this work and has approved it for publication.

## Funding

This work was supported by FORTE - Swedish Research Council for Health, Working Life and Welfare under Grant 2019-00066.

## Conflict of interest

The author declares that the research was conducted in the absence of any commercial or financial relationships that could be construed as a potential conflict of interest.
